# Correction: Genetic Pool Information Reflects Highly Suitable Areas: The Case of Two Parapatric Endangered Species of Tuco-tucos (Rodentia: Ctenomiydae)

**DOI:** 10.1371/journal.pone.0105485

**Published:** 2014-08-06

**Authors:** The PLOS ONE Staff

The word “Ctenomyidae” is misspelled in the article title. The correct title is: Genetic pool information reflects highly suitable areas: the case of two parapatric endangered species of tuco-tucos (Rodentia: Ctenomyidae). The correct citation is: Galiano D, Bernardo-Silva J, Freitas TROd (2014) Genetic Pool Information Reflects Highly Suitable Areas: The Case of Two Parapatric Endangered Species of Tuco-tucos (Rodentia: Ctenomyidae). PLoS ONE 9(5): e97301. doi:10.1371/journal.pone.0097301

There is an error in the fifth sentence of the first paragraph of the “Genetic data” segment of the Methods section. The correct sentence is: For C. lami, 27 locations were sampled, and the data are presented by the authors based on the four karyotypic blocks proposed for the species (Block A, B, C and D; see Freitas [4], Lopes and Freitas [19]).

There are errors in the first two sentences of the second paragraph of the “Genetic data” segment of the Methods section. The correct sentences are: Briefly, the geographical genetic structure of C. minutus was characterized by examining 276 individuals over the entire distributional range and using information from cytochrome oxidase I gene and control region sequences. For C. lami, a total of 166 specimens were sampled, using information from the same mitochondrial DNA markers.

The authors would like to add the following sentence to the Acknowledgments section: “We are grateful to Carla M. Lopes for suggestions of corrections to the published version of the manuscript.”
